# Characterization of trace metal content in the developing zebrafish embryo

**DOI:** 10.1371/journal.pone.0179318

**Published:** 2017-06-15

**Authors:** Rebecca T. Thomason, Michael A. Pettiglio, Carolina Herrera, Clara Kao, Jonathan D. Gitlin, Thomas B. Bartnikas

**Affiliations:** 1 University of Virginia, Charlottesville, Virginia, United States of America; 2 Department of Pathology and Laboratory Medicine, Brown University, Providence, Rhode Island, United States of America; 3 Eugene Bell Center, Marine Biological Laboratory, Woods Hole, Massachusetts, United States of America; CINVESTAV-IPN, MEXICO

## Abstract

Trace metals are essential for health but toxic when present in excess. The maintenance of trace metals at physiologic levels reflects both import and export by cells and absorption and excretion by organs. The mechanism by which this maintenance is achieved in vertebrate organisms is incompletely understood. To explore this, we chose zebrafish as our model organism, as they are amenable to both pharmacologic and genetic manipulation and comprise an ideal system for genetic screens and toxicological studies. To characterize trace metal content in developing zebrafish, we measured levels of three trace elements, copper, zinc, and manganese, from the oocyte stage to 30 days post-fertilization using inductively coupled plasma mass spectrometry. Our results indicate that metal levels are stable until zebrafish can acquire metals from the environment and imply that the early embryo relies on maternal contribution of metals to the oocyte. We also measured metal levels in bodies and yolks of embryos reared in presence and absence of the copper chelator neocuproine. All three metals exhibited different relative abundances between yolks and bodies of embryos. While neocuproine treatment led to an expected phenotype of copper deficiency, total copper levels were unaffected, indicating that measurement of total metal levels does not equate with measurement of biologically active metal levels. Overall, our data not only can be used in the design and execution of genetic, physiologic, and toxicologic studies but also has implications for the understanding of vertebrate metal homeostasis.

## Introduction

Trace metals such as copper (Cu), manganese (Mn), and zinc (Zn) are essential for health but toxic when present in excess [1]. This dual nature is best exemplified by inherited diseases of metal excess and deficiency. For example, Wilson disease is a disease of Cu excess that primarily affects the brain and liver and is caused by mutations in ATP7B, a metal transporter essential for hepatobiliary Cu excretion [2]. In contrast, Menkes disease is a disease of Cu deficiency characterized by multiple defects including impaired growth and neurologic dysfunction and is caused by mutations in ATP7A, a metal transporter essential for dietary Cu absorption and Cu delivery to the brain [2]. Three inherited forms of Mn excess or deficiency have been reported in recent years, although the underlying mechanism by which each gene defect leads to disease is not yet well understood. Mutations in the cellular Mn export protein SLC30A10 lead to Mn excess, dystonia, Parkinsonism, polycythemia, and chronic liver disease, while mutations in the cellular Mn import protein SLC39A14 lead to a similar disease albeit without polycythemia and liver disease [3,4]. Mutations in SLC39A8 lead to Mn deficiency, impaired development, and neurologic defects [5–7]. Inherited forms of Zn deficiency exist as well. Acrodermatitis enteropathica is a disease of dermatitis, alopecia, and diarrhea caused by mutations in SLC39A4, a gene essential for dietary Zn absorption. Transient neonatal zinc deficiency is a condition of dermatitis, alopecia, and impaired growth and immune function observed in infants breast-fed by mothers with mutations in SLC30A2, a transporter essential for establishing sufficient Zn levels in breast milk [8]. While genes essential for metal homeostasis have been studied in a variety of contexts other than inherited human disease, the nature of these human diseases highlights that metals must be maintained at sufficient but non-toxic levels for biological processes in the body and that metal homeostasis is achieved by a balance between import and export at both the cell and organ level.

Our understanding of the molecular basis of metal homeostasis has resulted, in part, from establishment and characterization of models of human disease and identification of essential genes using genetic screens in model organisms. A commonly used model organism is the zebrafish, a tractable experimental model that can be manipulated pharmacologically and genetically [9]. Its development occurs rapidly and can be readily observed. In this paper, we report the levels of three metals, copper (Cu), manganese (Mn), and zinc (Zn), in developing zebrafish. We chose to focus on zebrafish given their utility as a model organism and the fact that metal homeostasis in the developing organism is not thoroughly characterized. We chose to focus on Cu, Mn, and Zn as they vary in abundance in biological systems, with Zn the most abundant and Mn the least [10]. The results of our studies will not only be of use to investigators exploiting this model for studies of metal homeostasis but also have implications for our understanding of vertebrate metal homeostasis.

## Materials and methods

### Embryos and drug treatments

All zebrafish work was performed at the Marine Biological Laboratory and approved by the Institutional Animal Care and Use Committee under protocol number 16–36. Zebrafish (*Danio rerio*) embryos were collected from single pair matings and raised at 28.5°C in egg water (60 μg/ml Instant Ocean stock salts (Pentair) in system water) [11]. Adult fish were maintained in system water, produced by the fish facility by ultrafiltration, irradiation with ultraviolet light, and biofiltration. Zebrafish strains used in this study were AB (wild type). All embryos were staged by hours post-fertilization (hpf) and morphological criteria based on the zebrafish staging chart [12]. Non-fertilized oocytes were acquired from sexually mature female fish by anesthetizing the fish in Tricaine (4.2% 3-amino benzoic acidethylester) (Sigma) and squeezing until oocytes were released. The eggs were collected and placed in egg water and the female was returned to system water for recovery. For drug treatments, embryos were reared in 5 μM neocuproine (Sigma) in egg water at 28.5°C until the desired stage was reached. (While 10 μM was used in previous studies [13], our more recent work indicates that 5 μM is equally effective at inducing phenotypes of Cu deficiency (data not shown).) Control embryos were reared in egg water. 10 mM stock solutions of neocuproine were prepared in dimethyl sulfoxide (DMSO) (Sigma). Zebrafish were euthanized by Tricaine overdose.

### Sample processing and metal measurements

**Fig 1:**
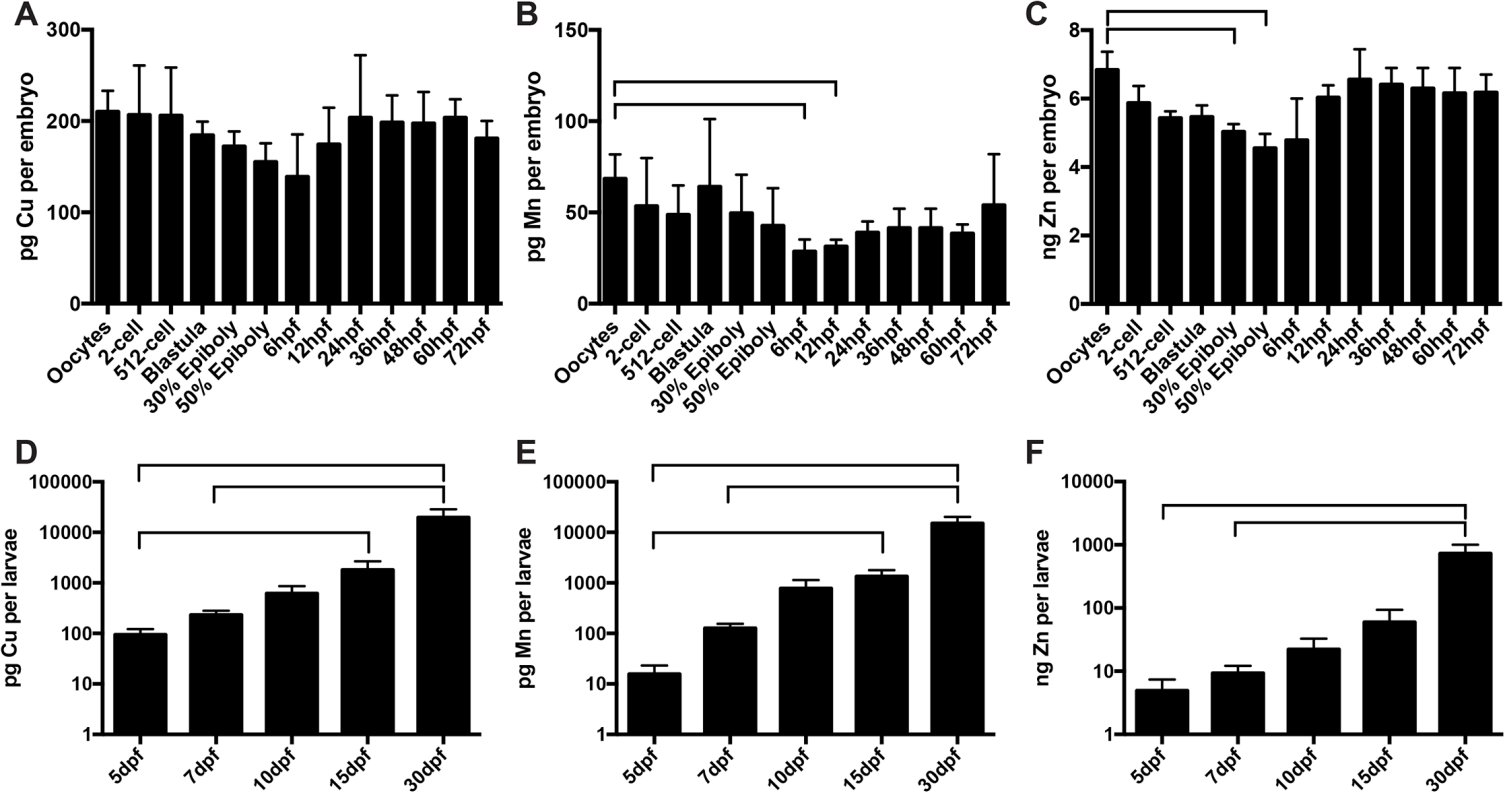
Metal levels in oocytes and embryonic and larval stages of zebrafish. ICP-MS measurements for copper (Cu) (A, D), manganese (Mn) (B, E), and zinc (Zn) (C, F) were performed on oocytes, embryos (2-cell, 512-cell, blastula, 30% epiboly, 50% epiboly, 6, 12, 24, 36, 48, 60, 72 hours post-fertilization (hpf)) (A-C) and larval fish (5, 7, 10, 15, 30 days post-fertilization (dpf)) (D-F). Each bar represents the average and standard deviation of four to five independently collected replicates for a specific developmental stage. Each replicate represents a pooled group of 20 oocytes, 10–20 embryos, or 5–10 larval fish. Metal levels are expressed per oocyte, embryo, or larva. Horizontal brackets above bars indicate a statistically significant difference between bracketed values. (For data in all panels, the Shapiro-Wilk normality test failed, therefore Kruskal-Wallis one-way ANOVA on ranks was performed, with Dunn’s method used for multiple comparison and P<0.05 as cut-off for significance.)

**Fig 2:**
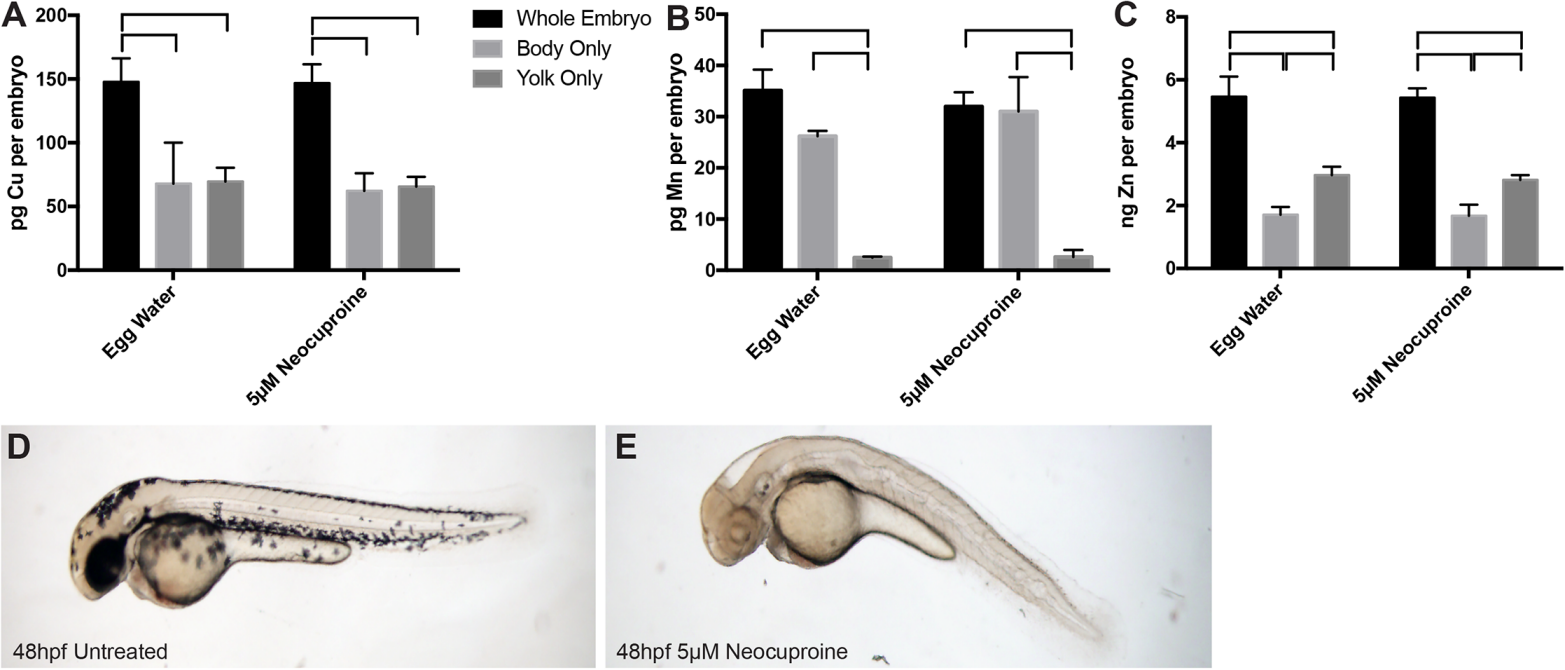
Distribution of metals in bodies and yolk sacs of embryos. (A-C) ICP-MS measurements were collected for copper (Cu) (A), manganese (Mn) (B), and zinc (Zn) (C) in whole embryos, bodies only, and yolk sacs only of embryos at 48 hpf, reared in presence or absence of 5 μM neocuproine, a Cu chelator. Each bar represents the average and standard deviation of three independently collected replicates, with each replicate consisting of a pooled group of 47–50 embryos. Horizontal brackets above bars indicate statistically significant difference between bracketed values. (Data in all panels passed Shapiro-Wilk normality and equal variance tests. One-way ANOVA indicated significant difference between groups, with the Holm-Sidak method used for multiple comparisons and P<0.05 as cut-off for significance.) There was no significant difference in metal levels between neocuproine-treated and–untreated samples of the same sample type. (D, E) Images were taken of zebrafish embryos at 48 hpf, reared in presence or absence of 5 μM neocuproine.

For all experiments, nitric acid (67–69%, Optima™, for ultra trace elemental analysis; Fisher Chemical) and water processed by MilliQ (Millipore) were used. All glassware and plasticware were washed three times in 10% nitric acid overnight and rinsed thoroughly with water after each acid wash. To collect samples, embryos (fertilized eggs to embryos up to 72 hpf) and larvae (5 to 30 days post-fertilization or dpf) were removed from egg or drug-treated water and placed in water in plastic petri-dishes on ice. Embryos and larvae were washed numerous times with cold water, pooled and placed in 1.5 mL polypropylene microcentrifuge tubes (Axygen), then flash frozen in liquid nitrogen and stored at -80°C until analysis. For data presented in Fig 1, one replicate consisted of 20 oocytes, 10–20 embryos, or 5–10 larval fish. For data presented in Fig 2, one replicate consisted of 47–50 pooled embryos. Analysis of pooled oocytes, embryos, or larva rather than individual organisms was performed to ensure that metal levels were above the lower limits of detection and levels of possible contaminating metals.

To digest samples for metal analysis, samples were thawed, resuspended in 200 μL nitric acid, and heated at 95°C until dry. Samples were then resuspended in nitric acid and dried again. Samples were resuspended in 200 μL hydrogen peroxide (30%, Optima™; Fisher Chemical) and heated at 95°C until dry. Samples were resuspended in hydrogen peroxide and heated until dry again. Samples were then resuspended in 1 mL 2% nitric acid. Mock digests, consisting of empty tubes, were processed identically as tubes containing zebrafish oocytes, embryos, or larva to account for any metal contamination during sample collection and processing. Approximately 50 samples were digested at a time, including three mock digests.

To analyze digested samples for metal levels, samples were analyzed by inductively coupled plasma mass spectrometry (ICP-MS) on an XSeries II spectrometer (Thermo Scientific) for copper (^65^Cu), zinc (^66^Zn), and manganese (^55^Mn) levels. At the beginning of each run, standards with known metal concentrations were analyzed to calibrate the instrument. Experimental samples, including mock digests, were then analyzed. Approximately 50 experimental samples were analyzed per run. After the analysis of five experimental samples, one of the standards was reanalyzed. This cycle was repeated until all experimental samples were analyzed. Each sample was analyzed by ICP-MS with a total of 300 sweeps, with the sweeps grouped into five sets of 60 sweeps. If the relative standard deviation across the five sets of sweeps was greater than 10%, the sample was reanalyzed. Instrument software was not used to calculate metal concentrations in each sample; rather, signal intensities measured for each standard and sample were analyzed using Excel as described immediately below. This approach allowed us to correct for changes in instrument sensitivity during each run, as could be detected by repeat analysis of standards throughout each run.

To determine the concentration of metals, a standard curve was plotted using the signal intensities and known metal concentrations in each standard, as analyzed at the beginning of each run. To account for metal contamination during sample processing, the average signal intensity in three mock digests was calculated and subtracted from the signal intensity measured for each experimental sample. The standard curve was then used to convert the mock-corrected signal intensities into metal concentrations. To account for changes in instrument sensitivity during each run, the signal intensities measured in repeat analyses of standard solutions throughout each run were plotted versus time of analysis. From this curve, correction factors, specific to the time that each experimental sample was analyzed, were determined and applied to the metal concentration calculated for each sample. Once the total metal levels in a specific sample were determined, the metal levels were normalized to number of individual organisms present in the original sample. All raw data, including metal levels in each replicate and mock digests, are included in supplemental data (S1 Fig). Sigmaplot was used for statistical analyses. Data shown in Fig 1 was analyzed by Shapiro-Wilk normality tests and one-way ANOVA on ranks, with Dunn’s method used for pairwise multiple comparisons. Data shown in Fig 2 was analyzed by Shapiro-Wilk normality and equal variance tests, followed by one-way ANOVA with the Holm-Sidak method used for pairwise multiple comparisons. Output from Sigmaplot analyses is included in supplemental data (S1 Fig).

To validate ICP-MS results, a small subset of samples were analyzed by graphite furnace atomic absorption spectrometry (GF-AAS) using an AAnalyst 600 spectrometer (Perkin Elmer). During GF-AAS runs, National Institutes of Standards and Technology certified reference material 1640a was measured to ensure run consistency.

## Results

To determine Cu, Mn, and Zn levels in developing zebrafish, oocytes, embryos, and larvae were digested in nitric acid and hydrogen peroxide and analyzed by ICP-MS. Zn was the most abundant metal, followed by Cu then Mn. Between the oocyte and 72 hpf stages, Mn levels decreased from oocyte to 6 and 12 hpf and Zn levels decreased from oocyte to 30% and 50% epiboly (Fig 1B and 1C). Between the 5 and 30 dpf stages, Cu and Mn levels increased from 5 to 15 dpf, 5 to 30 dpf, and 7 to 30 dpf while Zn levels increased from 5 to 30 dpf and 7 to 30 dpf (Fig 1D–1F).

To determine the relative abundance of metal in the bodies and yolks of zebrafish embryos, whole embryos, bodies, and yolks of zebrafish at 48 hpf were analyzed by ICP-MS. Cu was equally abundant in bodies and yolks, while Mn and Zn were more abundant in bodies and yolks respectively (Fig 2A–2C). Treatment of embryos with neocuproine, a cell-permeable Cu chelator, did not affect total metal levels even though it did cause loss in pigmentation and notochord defects, both of which are previously observed phenotypes of Cu deficiency [13] (Fig 2).

## Discussion

Our analyses of metal levels in developing zebrafish indicate that Zn is the most abundant metal followed by Cu then Mn, as has been observed in other biological systems [10]. The absolute metal levels and distribution between yolk and embryo that we ascertained by ICP-MS agree with most published reports. Using inductively coupled plasma optical emission spectroscopy, Ho et al. measured 7.5–10 ng Zn per embryo from 0 to 120 hpf [14]. We observed 4–7 ng Zn per embryo over the same stage of development (Fig 1C). Using X-ray fluorescence microtomography, Bourassa et al. noted that most Zn in the zebrafish embryo is contained within the yolk while Cu is distributed roughly evenly between yolk and body [15]. We observed similar patterns for both these metals (Fig 2A and 2C). However, using GF-AAS, Riggio et al. reported 10–75 ng Cu per embryo and 50–500 ng Zn per embryo from the oocyte stage to 5 hpf [16]. In contrast, we observed levels roughly one to two orders of magnitude lower, with 150–200 pg Cu per embryo and 4–7 ng Zn per embryo over the same developmental stage. Riggio et al. do not indicate the strain of zebrafish used in their study—they note only that sexually mature zebrafish were purchased from a local dealer—but it is unlikely that strain-specific differences explain the large difference in metal content reported in their study and ours. Riggio et al. also reported that Cu and Zn levels increased after fertilization, then decreased steadily over five hours after fertilization. In contrast, we observed that Cu and Zn levels were stable over this time period (Fig 1D and 1F). Ho et al. also observed that Zn levels were stable from 0 to 120 hpf [14]. At this time, we are unable to explain the discrepancy between our results and the results from Riggio et al. To control for differences in measurement techniques, we did analyze a small subset of digested samples by graphite furnace atomic absorption spectroscopy (GF-AAS). Metal levels measured by GF-AAS were on average 50% greater than those measured by ICP-MS (S1 Fig). This suggests that differences in measurement technique cannot account for the difference of one to two orders of magnitude between our results and those from Riggio et al.

As mentioned above, our analyses indicate that Cu, Mn, and Zn levels do not vary in early development and only increase once zebrafish develop to the stage where they can acquire metals from their environment. This suggests that either the early zebrafish does not exchange metals with its environment or that the rate of metal import into the organism is matched by the rate of export out of the organism. We favor the former, as it is a simpler explanation for a lack of change in metal content. This data also suggests that the maternal contribution to the oocyte sets the metal content of the early embryo. The implication of this notion is that perturbations in the biological processes that ensure appropriate metal levels in the oocyte could have severe effects on metal-dependent processes in developing progeny, as the early embryo cannot acquire metal from its environment. However, our results do not suggest that metal distribution within the organism itself is fixed. Our finding that Cu chelation does not affect the relative or absolute abundance of metals in bodies and yolks of embryos but does lead to pigmentation and notochord defects highlights the dynamic nature of Cu within the embryo. These results indicate that Cu can be rendered unavailable for processes such as melanin production by Cu-dependent tyrosinase without altering total levels within the embryo or distribution between yolk and body. This point may also be of interest to other investigators as it indicates that measurement of total metal levels does not equate with measurement of biologically active or available metals. Another finding that supports the notion that metal distribution within the embryo is dynamic even if the embryo is not importing metals from or exporting metals into its environment is that morpholino antisense knockdown of Slc39a7/Zip7, a member of a solute carrier family implicated in Zn transport, alters the distribution of Zn without altering total Zn content in zebrafish embryos [17].

The asymmetric distribution of metals between yolk and embryo and, as demonstrated by microtomography by Bourassa et al. [15], throughout the embryo itself may be due, at least in part, to differential expression of metal transporters throughout the embryo. This possibility is supported by expression studies of Slc39a/Zip and Slc30a/Znt, two families of transporters with known or presumed roles in Zn transport. Yan et al. demonstrated that expression of Slc39a/Zip genes 1, 3, 4, 6–11, and 13 and Slc30a/Znt genes 1, 2, and 4–9 varied significantly across the 0–120 hpf period. If these changes in transporter gene expression have functional relevance, they do not impact total Zn levels in the early embryo. This concept is also relevant to Mn, as Slc39a8/Zip8 has recently been clinically implicated in cellular Mn import, although its precise role has yet to be established. Individuals with SLC39A8/ZIP8 mutations develop cranial asymmetry, infantile spasms, and dwarfism, along with undetectable blood manganese levels and severe deglycosylation of blood proteins [18]. This clinical picture is attributed to impaired cellular Mn import leading to impaired activity of Mn-dependent enzymes critical for biosynthesis of glycoproteins. Notably, Yan et al. observed detectable expression levels of Slc39a8/Zip8 only by 12 hpf which then increased up to 120 hpf, the last time point measured in their experiment. In our study, Mn levels do not vary in the early embryo. If changes in Slc39a8/Zip8 expression have functional relevance, they may impact Mn distribution within the embryo without altering total Mn levels.

Besides the aforementioned physiologic implications, our studies have several technical implications. Zebrafish can be used for genetic screens to identify molecular determinants of the metabolism of Cu and other metals [19] and can also be employed as a model system for studying toxicity of environmental exposure to essential metals [20]. A recent study by Bakthavatsalam et al. established zebrafish as a potent model to study neurotoxicity induced by environmental Mn exposure [21]. Our study establishes the baseline total levels of three essential trace elements in zebrafish embryos and larvae which can be used as reference when designing and interpreting screens for genes required for metal homeostasis and studies of acquired metal toxicity. Furthermore, the stability of metal levels we observed in the early zebrafish embryo suggests that inherited defects may not manifest as changes in total metal levels until the zebrafish is adequately developed to exchange metals with its environment. Another point to consider is that maternal expression of genes contributes to the transcriptome of early zebrafish, which may mask deficiencies in zygotic expression of genes relevant to metal homeostasis.

## Conclusions

Our observation that levels of trace elements Cu, Mn, and Zn increased only in zebrafish at the larval stage suggest that embryonic metal levels are set by the maternal contribution to the oocyte and that metal levels increase in zebrafish once the organism is able to acquire nutrients from its environment. These findings can be used not only to plan, execute, and interpret genetic and toxicology studies in zebrafish but they also underscore the essential role of the maternal environment in meeting the nutritional requirements of the early vertebrate embryo.

## Supporting information

S1 Fig Raw data for metal analyses with revised statistics. This Excel file, entitled “raw data for metal analyses revised stats”, contains three worksheets. The first and second worksheets contain raw data and statistical analyses for data presented in Figs 1 and 2. The third worksheet contains raw data on the comparison of metal measurements in a subset of zebrafish samples by GFAAS and ICPMS.
(XLSX)
